# Overexpression of *BASP1* Indicates a Poor Prognosis in Head and Neck Squamous Cell Carcinoma

**DOI:** 10.31557/APJCP.2020.21.11.3435

**Published:** 2020-11

**Authors:** Ashwin Jaikumar Ram, Smiline Girija AS, Vijayashree Priyadharsini Jayaseelan, Paramasivam Arumugam

**Affiliations:** 1 *Department of Microbiology, Saveetha Dental College, Saveetha Institute of Medical and Technical Sciences, Saveetha University, Chennai, India. *; 2 *BRULAC-DRC, Saveetha Dental College, Saveetha Institute of Medical and Technical Sciences, Saveetha University, Chennai, India. *

**Keywords:** Head and neck squamous cell carcinomas (HNSCC), BASP1, prognostic marker

## Abstract

**Objective:**

Brain abundant membrane attached signal protein 1 (*BASP1*) was originally identified as a membrane and cytoplasmic protein. Recent studies have shown that *BASP1* highly expressed in cancer and promoted the proliferation of cancer. However, the role of *BASP1* in head and neck squamous cell carcinoma (HNSCC) is largely unknown. Here, we performed a systematic data analysis to examine whether *BASP1* can function as prognostic marker in HNSCC.

**Methods:**

In this study, we used Oncomine, and UALCAN, databases to analyze the expression of *BASP1* in HNSCC. We used Kaplan-Meier plotter to evaluate the effect of *BASP1* on clinical prognosis. In addition, we also analyzed genetic alterations, interaction network, and functional enrichment of *BASP1*.

**Results:**

*BASP1* mRNA expression level was remarkably increased in HNSCC than in normal tissues (P=1.624e-12). Moreover, high *BASP1* expression was significantly related to poor survival (p=0.00056) in HNSCC patients. In addition, *BASP1* gene amplified in 5% of HNSCC patients which contributes to the overexpression of *BASP1*.

**Conclusions:**

These findings suggest that *BASP1* was frequently amplified which contributes to the overexpression of *BASP1*, thereby promoting HNSCC progression. Thus, these results indicate that *BASP1* might serve as a biomarker to predict the progression and prognosis of HNSCC patients.

## Introduction

Head and neck squamous cell carcinoma (HNSCC), a common malignant tumor of the head and neck distinct, which arises from lip, oral cavity, paranasal sinuses, oropharynx, larynx, nasopharynx and other pharynx carcinomas (Xiao-Nan et al., 2018). As the sixth most common type of malignant tumor with an incidence of over 650,000 new cases and a 90,000 deaths per year worldwide (Torre et al., 2012). Currently, cigarette smoking, alcohol consumption as well as human papilloma virus (HPV) infection are deemed to be risk factors for the occurrence and prognosis of HNSCC (Magnes et al., 2017). HNSCC account for 30% of all cancers in India. In north eastern India, tobacco-related cancers are very common because of the widespread use of tobacco (Sharma1 et al., 2019). Unfortunately, due to lack of symptoms in the early stage when detected of HNSCC is usually made at advanced stages and the 5-year survival rate is still under 50% now, while due to local recurrence and metastasis, which reduces survival rate to 35% (Chin et al., 2005). The occurrences and progression of HNSCC is a complicated process involving multiple molecules. Thus, to identify essential genes that could serve as effective biomarkers and potential treatment targets are urgently needed for HNSCC.

*BASP1* was initially identified as an abundant membrane bound protein in the brain and was thought to play a role in axon regeneration and neuronal plasticity (Maekawa et al., 1993; Bomze et al., 2001; Korshunova et al., 2008). *BASP1* has also been found in other cell types and at other subcellular locations, and its functions in transcriptional regulation, apoptosis, and differentiation have also been recently reported (Carpenter et al., 2004; Sanchez-Nino et al., 2010; Green et al., 2009). The *BASP1* gene was silenced in several tumour types including hepatocellular carcinoma, thyroid cancer and leukaemias (Yeoh et al., 2002; Moribe et al., 2008; Guo et al., 2016). Furthermore, cellular transformation by v-myc requires silencing of the *BASP1* gene (Hartl et al., 2009). More recent study demonstrated that *BASP1* highly expressed in cervical cancer that promoted cancer growth (Tang et al., 2017).

In recent years, studies have shown that abnormal expression and methylation status of the *BASP1* gene are closely related to the occurrence and prognosis of certain tumors and that *BASP1* may also play a role as a tumor suppressor gene. Zhou et al., (2018) recently reported that methylation-associated silencing of *BASP1* contributes to leukemogenesis in acute myeloid leukemia (AML). *BASP1* gene methylation and its expression level are also associated with melanoma progression (Ransohoff et al., 2017). However, the role of *BASP1* in HNSCC has not been reported.

In this study, we analyzed the relationship between *BASP1* expression and clinicopathological parameters in patients with HNSCC, and further studied the role of *BASP1* in the HNSCC patients survival. We found that *BASP1* is a new prognostic marker for HNSCC.

## Materials and Methods

*ONCOMINE Analysis*

ONCOMINE gene expression array datasets (https://www.oncomine.org/), an online cancer microarray database, is used to analyze the mRNA levels of the *BASP1* in different cancers. The mRNA expressions of the *BASP1* in clinical cancer specimens were compared with that in normal controls, using a Student’s t test to generate a p value. The cutoff of p value and fold change were defined as 0.01 and 2, respectively. 

*UALCAN Dataset*

Analysis of *BASP1* mRNA expression was carried out using UALCAN. UALCAN (http://ualcan.path.uab.edu) is a user-friendly, intelligence web asset for analyzing, integrating and discovering cancer transcriptome data and in-depth analyses of TCGA gene expression information (Chandrashekar et al., 2017).

*Analysis of c-BioPortal, STRING and Metascape Databases*

The cBioPortal (http://cbioportal.org) is an open-access asset gives visualization, analysis and download of substantial scale cancer genomics data sets which portal currently containing 245 cancer studies. We utilized c-BioPortal to analyze *BASP1* alterations and expression in the TCGA HNSCC samples (Cerami et al., 2012). We also used STRING database (https://string-db.org/) (Szklarczyk et al.,2016) to search protein–protein interaction (PPI) network for *BASP1* and Metascape (http://metascape.org) (Warde-Farley et al., 2010) to analyse the pathway and process enrichment of *BASP1*.

**Figure 1:**
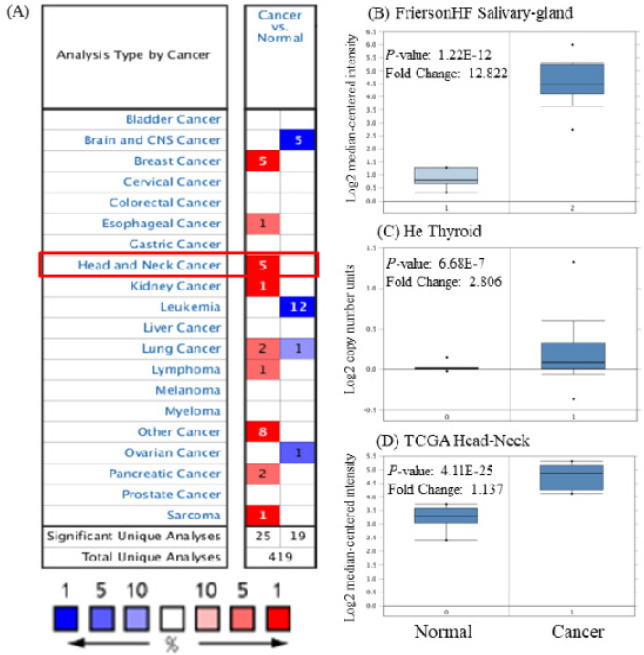
Expression of* BASP1*in Various Types of Cancers Using on the Oncomine. (A) mRNA expression of *BASP1 *in different types of cancers compared with normal tissues (red, overexpression; blue, downregulation). *BASP1 *mRNA levels in FriersonHF Salivary-gland (B), He Thyroid (C) and TCGA Head-Neck (D) grouped by HNSCC and normal tissue in the Oncomine database

**Figure 2:**
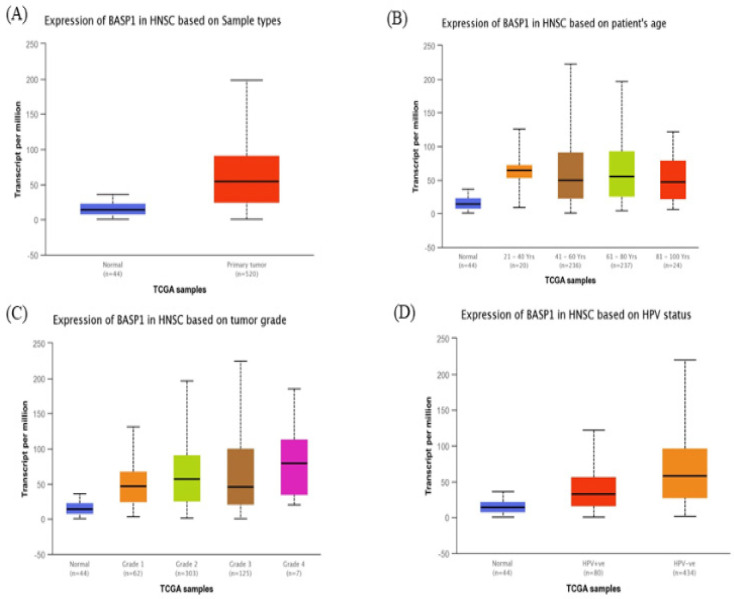
Boxplots Showing Relative Expression of *BASP1 *in Tumors Based on Sample Types, Age, Tumor Grade, and HPV Status via UALCAN Database (P < 0.05). (A) The boxplot shows the relative expression of *BASP1* in normal and HNSCC samples. (B) Expression of *BASP1* in HNSCC based on age. (C) Expression of *BASP1* in HNSCC based on tumor grade. (D) Expression of *BASP1* in HNSCC based on HPV status

**Figure 3:**
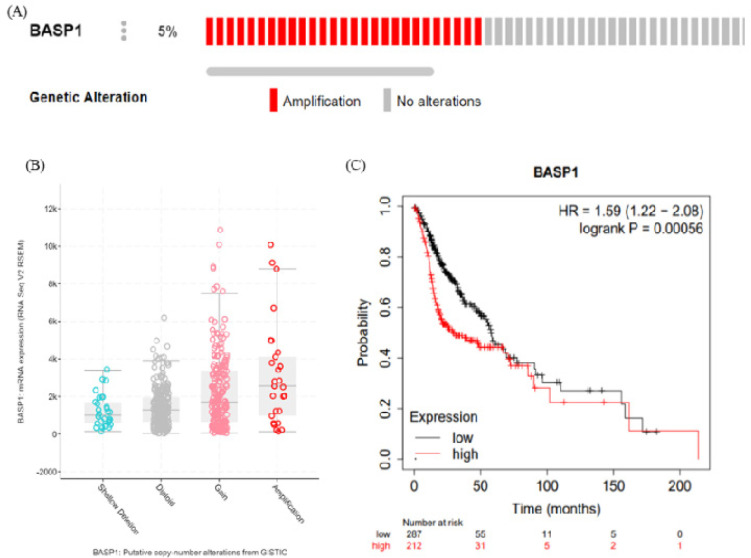
Correlation between the Genetic Alterations of *BASP1* and mRNA levels in HNSCC Tissues. Oncoprint in cBioPortal database exhibited the proportion and distribution of specimens with genetic alterations in *BASP1* (A). Copy gain (gain and amplification) of *BASP1 *was associated with notably increased BASP1 mRNA levels compared with the copy-neutral (diploid) and copy-loss (shallow deletion) cases (B). Kaplan-Meier curve revealed that upregulation of *BASP1* was correlated with significantly worse prognosis in HNSCC patients (p = 0.00056) (C)

**Figure 4:**
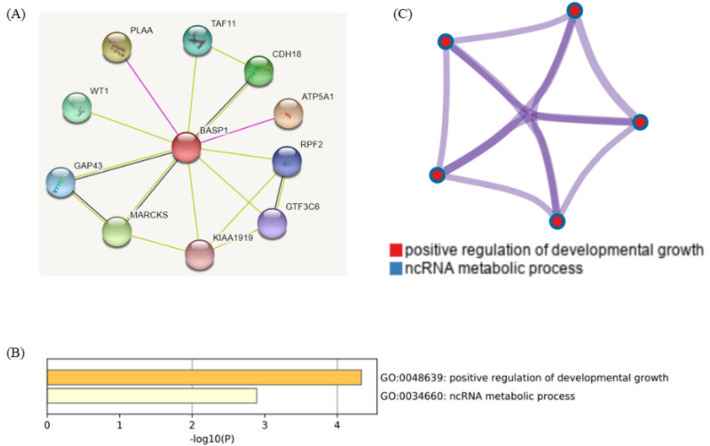
(A) Protein–protein interaction network between *BASP1*and its targets in the STRING dataset. (B, C) KEGG (Kyoto Encyclopedia of Genes and Genomes) pathway analysis of the *BASP1* through online website Metascape

## Results

*BASP1 is up-regulated in HNSCC*

Oncomine database analysis revealed that *BASP1* mRNA was increased in most types of cancers, including HNSCC, breast cancer, kidney cancer, pancreatic cancer, and lung cancer (Figure 1A). Box plot showing *BASP1* mRNA levels were significantly higher in HNSCC tissues (Figure 1B-D). Further subgroup analysis of multiple clinic pathological features of 520 HNSCC samples in the TCGA reliably indicated high transcription of *BASP1* (Figure 2). In age subgroup (normal-vs-age (21–40 yrs), normal-vs-age (41–60 yrs), normal-vs-age (61–80 yrs) and normal-vs-age (81–100 yrs)) analysis the mRNA level of *BASP1* was essentially higher in HNSCC patients than healthy individuals; tumor grade subgroup (normal-vs- Grade 1, normal-vs-Grade 2, normal-vs-Grade 3, Grade 1-vs-Grade 2, Grade 1-vs-Grade 3, Grade 1-vs-Grade 4, Grade 2-vs-Grade 4 and Grade 3-vs-Grade 4) analysis the *BASP1* was also significantly higher in HNSCC patients (Figure 2). In HPV status subgroups (normal-vs-HPV – ve and normal-vs-HPV + ve) analysis the *BASP1* was also significantly higher in HNSCC patients (Figure 2).

*BASP1 gene amplification in HNSCC samples*

We analysed the genetic alterations and expression levels of *BASP1* by using the cBioPortal TCGA dataset. We found that *BASP1* gene was most frequently amplified (Figure 3A). Further, amplification of *BASP1* was positively correlated with its mRNA expression in HNSCC patients (Figure 3B). Moreover, overexpression of *BASP1* correlated with the poor prognosis of HNSCC patients (p=0.00056) (Figure 3C). 

*PPI and functional enrichment analysis of BASP1*

PPI analyses revealed that the *BASP1* interact with *PLAA, TAF11, CDH18, ATP5A1, RPF2, GTF3C6, KIAA1919, MARCKS, GAP43*, and *WT1* (Figure 4A). To study the functions of *BASP1*, we analyzed GO and KEGG pathways using Metascape. The result showed top 2 enrichment, molecular functions including positive regulation of developmental growth and non-coding RNA (ncRNA) metabolic process (Figure 4B and 4C). 

## Discussion

In the present study, we revealed that *BASP1* plays an important role in HNSCC. Investigation of transcriptional sequencing information from thousands clinical samples TCGA databases comprising six geographic regions and subgroups analysis stratified based on gender, age, HPV status, gender, race and tumor grade confirmed that *BASP1* mRNA levels and Copy number variations (CNVs) are fundamentally higher in HNSCC when compare with normal tissue. The highly expressed *BASP1* is a novel unfavourable prognostic factor for patients with HNSCC. Moreover, high *BASP1* levels correlated with poor clinical outcome. These results suggest that *BASP1* serves as a prognostic marker. More recent study also showed that *BASP1* was highly expressed in cervical cancer and plays an oncogenic role (Tang et al., 2017), 

However, previous reports have shown that *BASP1* is downregulated in v-myc-induced transformed cells, and that overexpression of *BASP1* inhibits transformation, further analysis showed that *BASP1* inhibits the target genes of c-Myc, such as WS5, Q83 and BRAK, suggested that *BASP1* could be a tumor suppressor (Hartl et al., 2009). Moribe and colleagues used a gene microarray and pyrosequencing to screen genes that are methylated specifically in hepatocellular carcinoma (HCC). They found that *BASP1* was aberrantly methylated in HCC, thereby its expression was reduced in HCC, and suggested that it can function as a useful biomarker for the diagnosis of HCC (Moribe et al., 2008). MicroRNA miR-191, an onco-miR, was upregulated in transformed human bronchial epithelial cells, and promotes epithelial-mesenchymal transition (EMT) and self-renewal of cancer stem cells of transformed cells. *BASP1* is a direct target of miR-191 and *BASP1* inhibition by miR-191 leads to transactivation of WT1, which activates the Wnt pathway to promote tumor progression (Xu et al., 2015).

Many genes have been found to play different roles in different kinds of tumors. For example, inhibitor of DNA binding 2 (ID2) is downregulated in breast cancer, in which it inhibits cellular invasion and associated with favorable prognostic factor for patients (Stighall et al., 2005). Other studies have also reported that ID2 is upregulated in brain cancer, colon cancer, pancreatic cancer, and prostate cancer, in which it promotes tumor progression, making it an unfavorable prognostic factor (Niola et al., 2013; Wilson et al., 2001; Maruyama et al., 1999; Coppe et al., 2004). However, few studies have reported the *BASP1* is downregulated in some cancer, but we found that *BASP1* is up-regulated in many cancers including HNSCC, breast cancer, esophageal cancer, kidney cancer, lung cancer, lymphoma, pancreatic cancer, and sarcoma. 

Moreover, we studied the functional enrichment and the mechanism of the *BASP1*. Our results demonstrated that the pathways involved in *BASP1* might include positive regulation of developmental growth and ncRNA metabolic pathway. Recent studies reported that these pathways play important roles in tumor growth and metastasis. Therefore, these findings help to study the roles of *BASP1* and relevant signaling pathways in HNSCC development and progression. 

In conclusion, the recent studies reported that gene copy number gain was most common genetic alterations and correlated with high expression, which promoted development of cancers. Importantly, we reported for the first time that *BASP1* copy number gain was striking higher which contributes to the overexpression of *BASP1*, thereby promoting HNSCC progression. Therefore, *BASP1* could be a promising prognostic biomarker and potential therapeutic target for HNSCC. 
